# Supplementary material for: Stress-induced phospho-ubiquitin formation causes parkin degradation
Source: Sci Rep. 2019 Aug 12;9:11682. doi: 10.1038/s41598-019-47952-5 (PMC6690910; doi:10.1038/s41598-019-47952-5)

Stress-induced phospho-ubiquitin formation causes parkin degradation

Lyudmila Kovalchuke[1], Eugene V. Mosharov[2], Oren A. Levy[3], Lloyd A. Greene[4]*

[1] Graduate Program in Biological Sciences, Columbia University, New York, NY, USA
[2] Departments of Psychiatry, Neurology, and Pharmacology, Columbia University: Division of Molecular Therapeutics, New York State Psychiatric Institute, New York, NY, USA
[3] Department of Neurology, Columbia University Medical Center, New York, NY, USA
[4] Department of Pathology and Cell Biology, Columbia University Medical Center, New York, NY, USA
* Corresponding author: lag3@cumc.columbia.edu

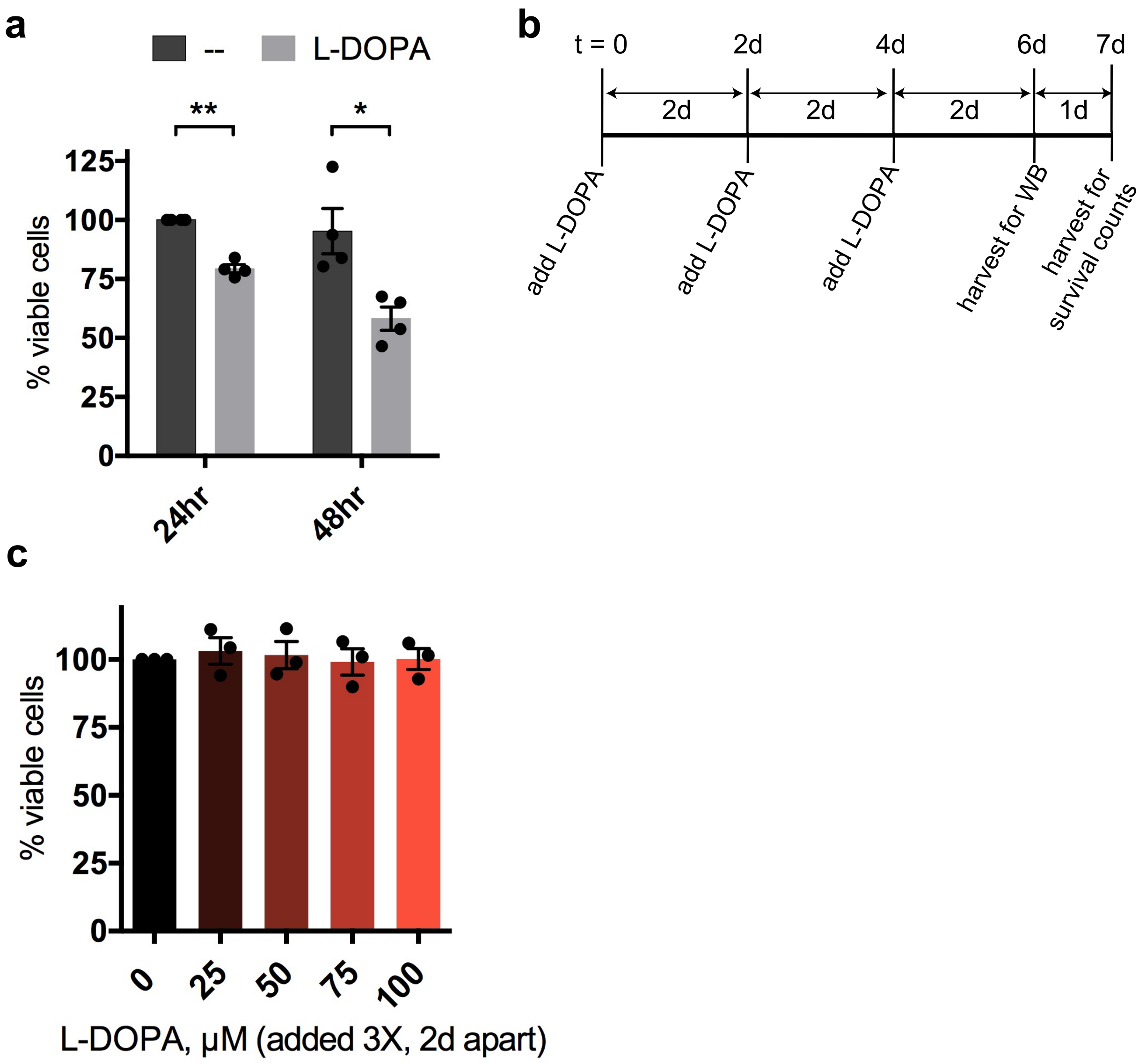

<u>Supplementary Figure 1. L-DOPA-induced parkin protein loss occurs independently of cell death</u>

A. Timecourse of L-DOPA toxicity in differentiated PC12 cells. Cells were treated with 200 µM L-DOPA and harvested for assessment of survival after 24 and 48 hours. B. Treatment paradigm used to assess whether non-toxic doses of L-DOPA lead to parkin loss. WB=Western immunoblot. C. Quantification of cell survival after exposure to L-DOPA following the paradigm in (B). A,C. Error bars show SEM from N = 4 (A) or 3 (C) independent experiments ; * $p \leq 0.05$, ** $p \leq 0.01$ by paired t-test with Holm correction for multiple comparisons.

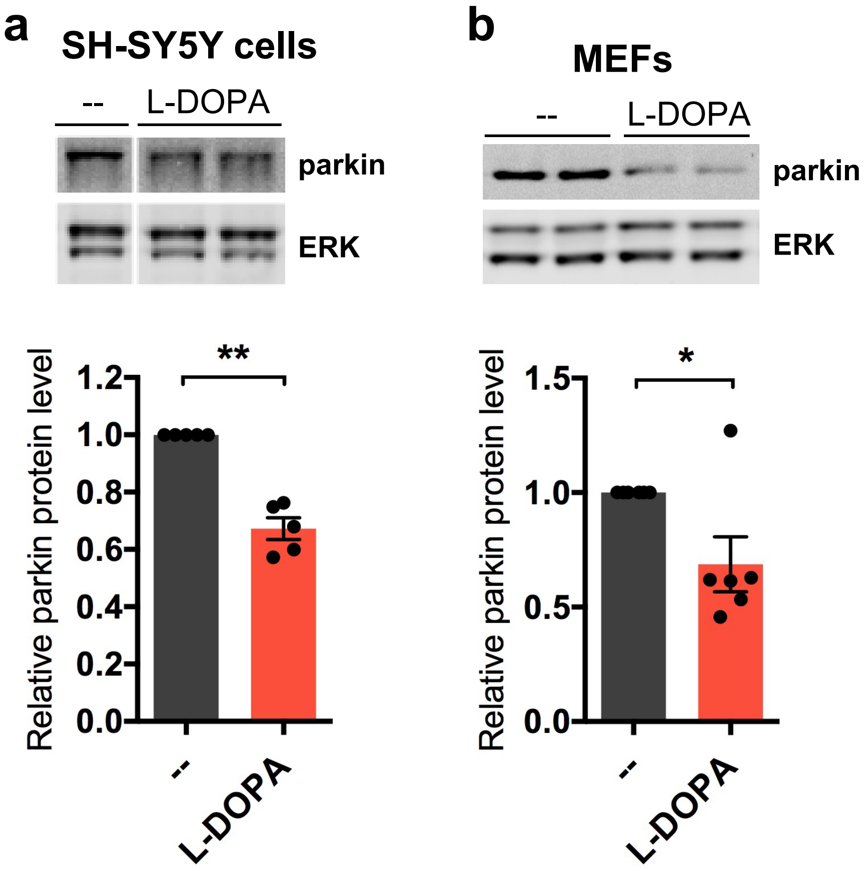

Supplementary Figure 2. L-DOPA treatment induces parkin protein loss in SH-SY5Y cells and mouse embryonic fibroblasts

L-DOPA induces parkin loss in undifferentiated SH-SY5Y cells (A) and immortalized MEFs (B). Cells were treated with 200 μM L-DOPA for 24 hours before being harvested for Western immunoblotting. Error bars show SEM from N = 5 (A) or 6 (B) independent experiments; * $p \leq 0.05$ by paired t-test.

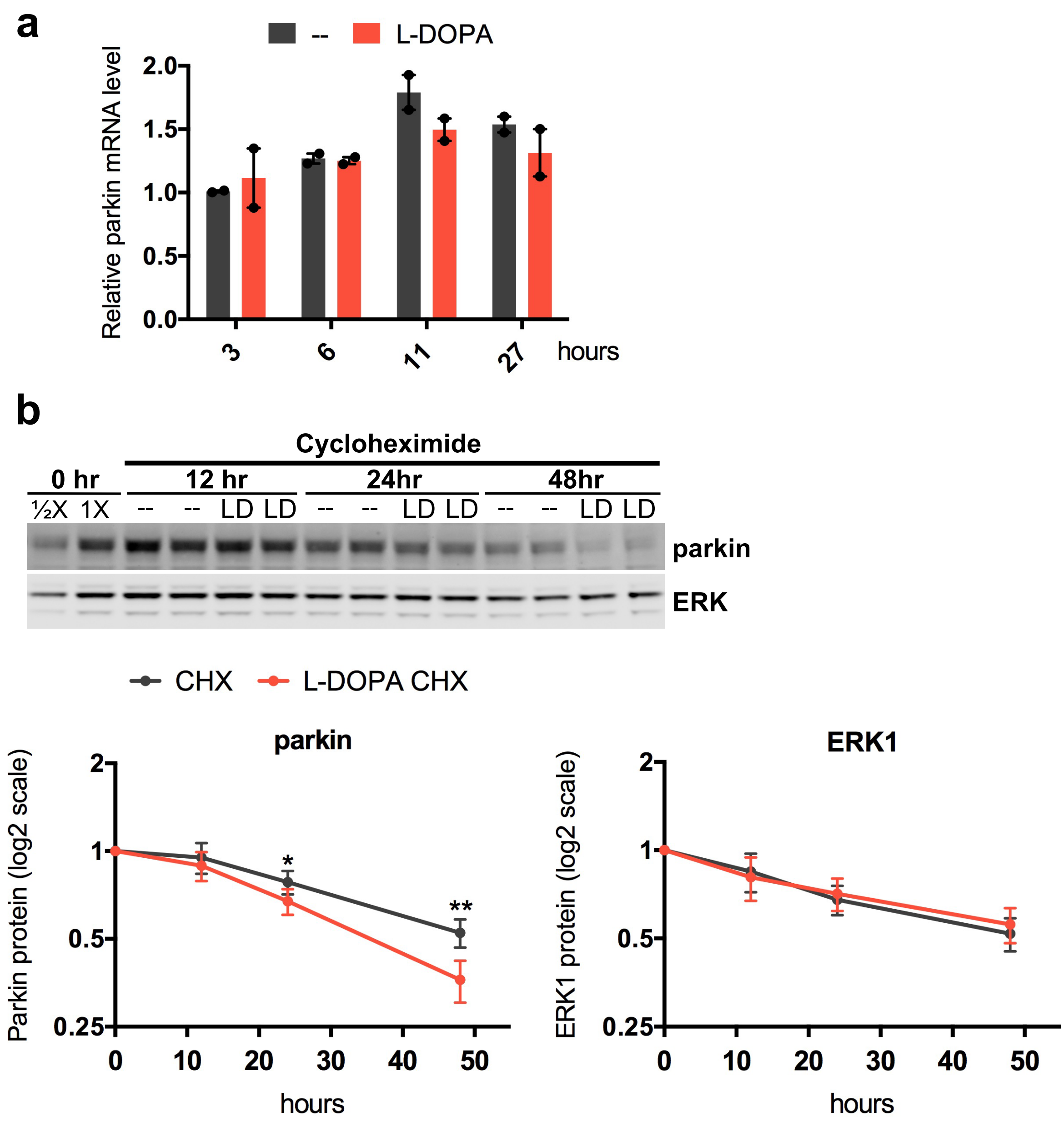

Supplementary Figure 3. L-DOPA treatment increases the rate of parkin protein turnover

A. L-DOPA does not alter parkin mRNA levels. Cells were treated with 200 µM L-DOPA and harvested for qPCR analysis at the indicated time points. The parkin mRNA levels were normalized to 18S ribosomal RNA. B. L-DOPA accelerates parkin loss in PC12 cells when translation is inhibited. Cells were treated with 2 µg/mL cycloheximide (CHX) in the presence or absence of 200 µM L-DOPA (LD) and harvested for Western blotting at the indicated time points. Representative Western blot and quantification of parkin and ERK1 levels not normalized to a loading control. Error bars show range from N = 2 independent experiments for (A) and SEM from N = 3 independent experiments for B; For A, significance was assessed at each time point by paired t-test followed by Holm correction for multiple comparisons. For B, * $p \leq 0.05$, ** $p \leq 0.01$ relative to no L-DOPA at that time point by repeated measures 2-way ANOVA of data from the 12-48 hour time points with Sidak's multiple comparisons test.

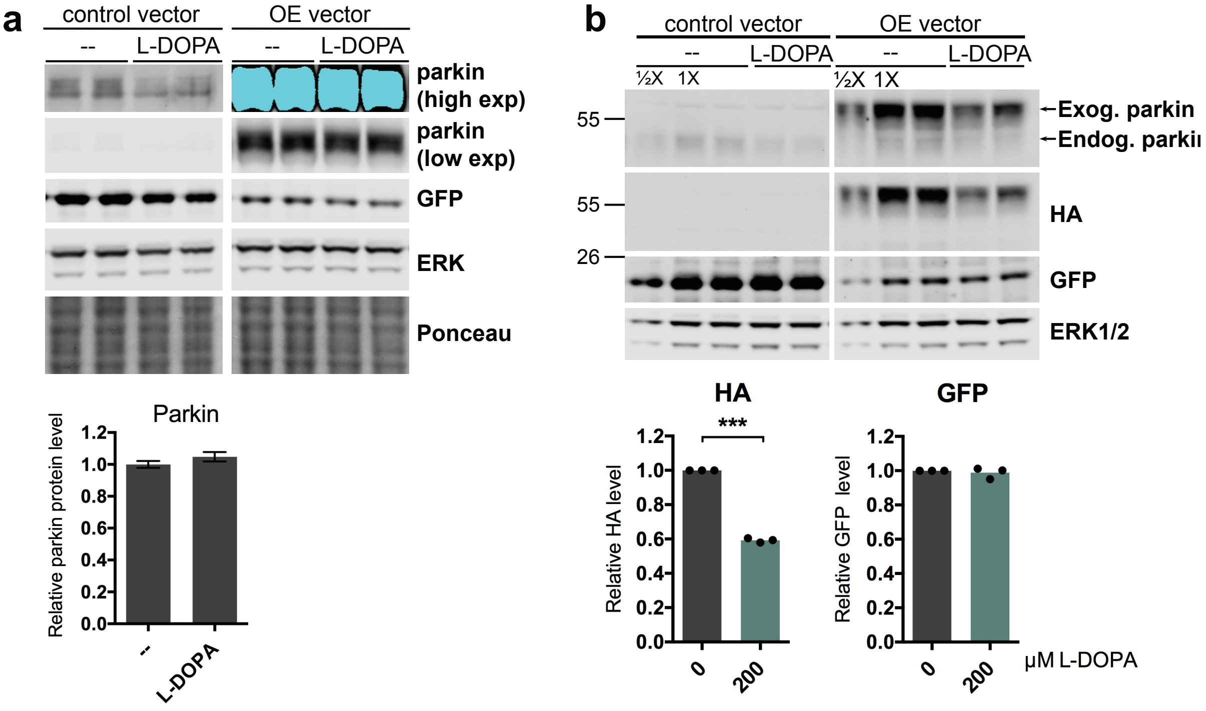

Supplementary Figure 4. L-DOPA treatment decreases moderately overexpressed parkin protein

A. Differentiated PC12 cells were transduced with a lentiviral vector carrying untagged rat parkin expressed from the EF-1α promoter (OE vector). GFP is expressed bicistronically with parkin using an IRES sequence. 16 hours after transduction, cells were treated with 200 µM L-DOPA and harvested for WB 48 hours later. Parkin protein levels were quantified relative to the average of ERK1 and Ponceau stain signals. Error bars in B show the range of the two biological replicates shown in the immunoblot. Results are representative of 2 experiments. B. Differentiated PC12 cells were transduced with a lentiviral vector carrying "moderately" overexpressed, N-terminally HA- and FLAG-tagged rat parkin expressed from the minimal human parkin promoter (OE vector). GFP is expressed bicistronically with parkin using an IRES sequence. 3 days after transduction, cells were treated with 200 µM L- DOPA for 24 hours and harvested for WB. Overexpressed parkin protein levels were detected using antibodies against parkin and HA. Quantification of overexpressed parkin and GFP levels with and without 200 µM L-DOPA treatment from N = 3 independent experiments is shown. Proteins were normalized to the average of ERK1 and 2. Error bars showing SEM are too small to be seen. *** $p \leq 0.001$ by paired t-test. A,B. Control vector was an empty version of the parkin construct used in A.

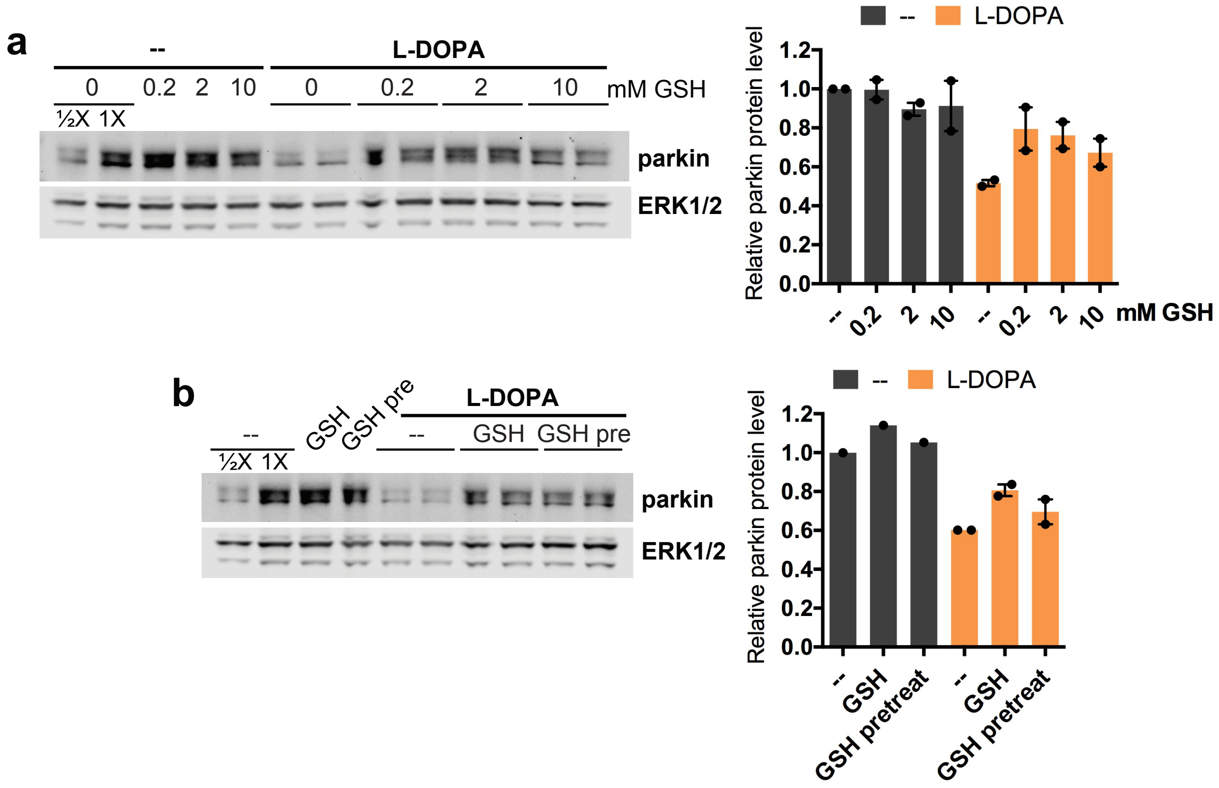

<u>Supplementary Figure 5. Effect of high glutathione doses and glutathione pre-treatment on L-DOPA-induced parkin loss</u>

A. Differentiated PC12 cells were co-treated with 200 µM L-DOPA and the indicated glutathione (GSH) concentrations for 24 hours before lysates were harvested for Western blotting. A representative Western blot and quantification of parkin levels from 2 independent experiments are shown. B. PC12 cells were co-treated with 200 µM L-DOPA and 200 µM GSH or pre-treated with 200 µM GSH for 4 hours prior to addition of L-DOPA ("GSH pre"). Data points in the quantification of parkin levels represent biological replicates from one experiment. Error bars show range.

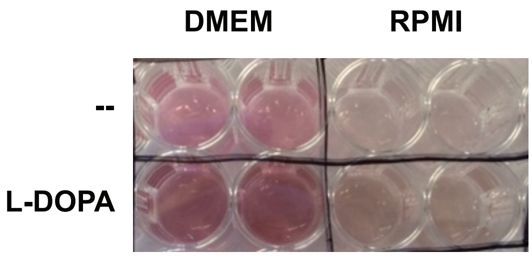

Supplementary Figure 6. L-DOPA causes browning of cell culture medium

200 μM L-DOPA was added to DMEM or RPMI culture medium and incubated for 24 hours at 37°C under 7.5% CO_2 in the absence of cells. Evidence of L-DOPA-derived quinones can be seen in the "browning" of the medium.

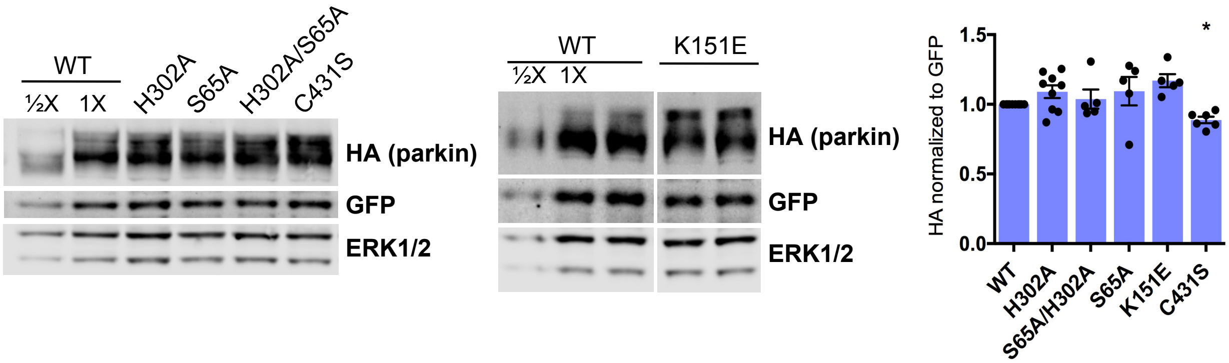

Supplementary Figure 7. Expression levels of parkin mutants are similar to one another

Differentiated PC12 cells were transduced for 3 to 6 days with lentiviral vectors carrying the indicated parkin mutants and bicistronically expressed GFP before cells were harvested for Western immunoblotting. Representative blots are shown. To compare relative expression levels of the different parkin mutants, parkin levels for each construct were normalized first to ERK1 and 2 to control for loading differences and then to GFP to control for variability in viral titer. The results of this quantification are shown on the right. Error bars show SEM from 5-10 independent experiments; * $p \leq 0.05$ relative to WT by paired t-test with Holm correction for multiple comparisons.

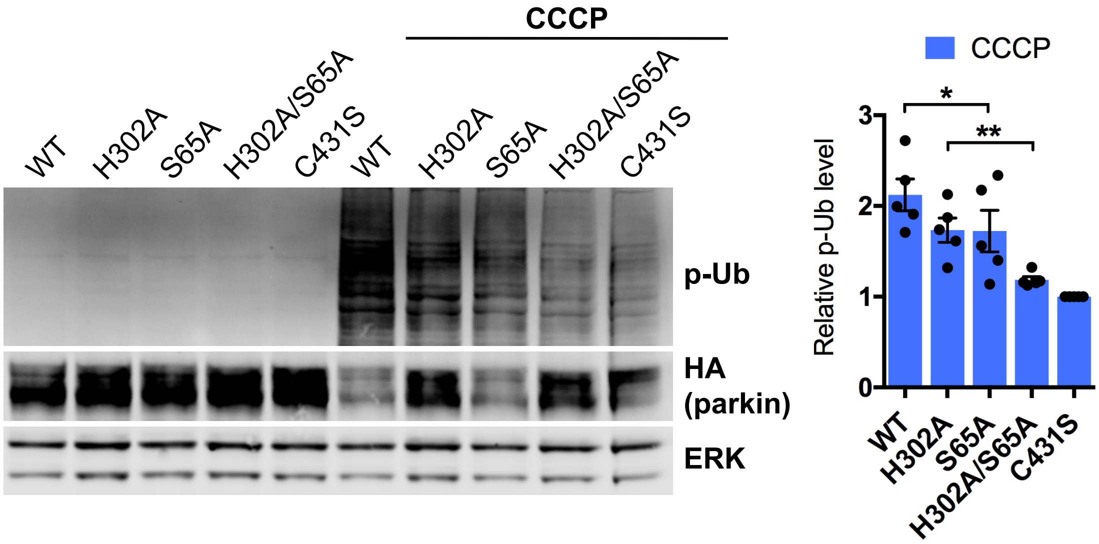

Supplementary Figure 8. Parkin phosphorylation plays a functional role in the CCCP model

Differentiated PC12 cells were transduced with indicated mutant parkin constructs and treated with CCCP for 6 hours as described in Fig. 5. Relative levels of phospho-poly-Ub for each mutant were quantified by normalizing the p-Ub signal first to ERK1/2 and then to the corresponding mutant parkin level in untreated cells as determined by probing with anti-HA (to control for infection efficiency). C431S is catalytically inactive parkin. Quantification from N = 5 independent experiments is shown. Error bars show SEM. * $p \leq 0.05$, ** $p \leq 0.01$ by paired t-test with Holm correction for multiple comparisons.

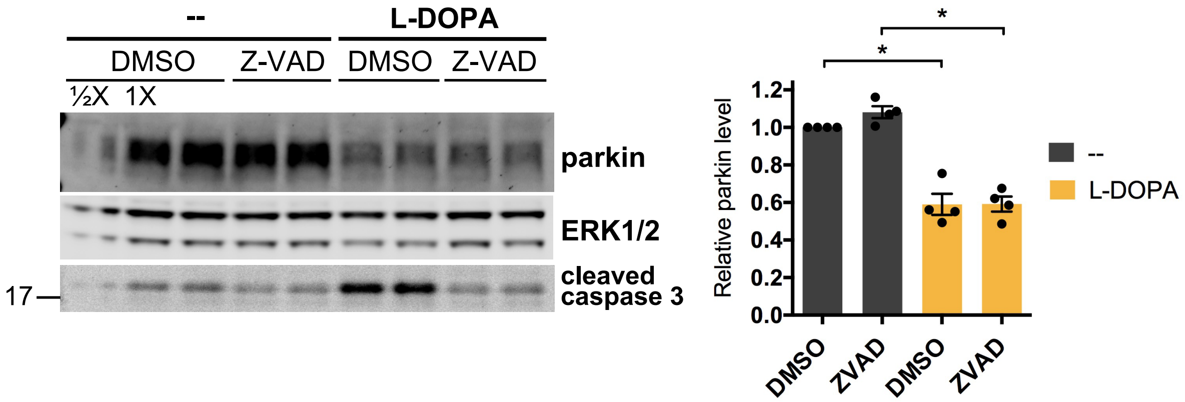

Supplementary Figure 9. L-DOPA-induced parkin loss is not attenuated by caspase inhibition

Differentiated PC12 cells were co-treated with 200 µM L-DOPA and 100 µM Z-VAD-FMK (Z-VAD) for 24 hours before cells were harvested for Western immunoblotting. Prevention of the formation of cleaved caspase 3 was a positive control for the effectiveness of Z-VAD-FMK. A representative blot and quantification of N = 4 independent experiments are shown. Error bars show SEM; * $p \leq 0.05$ by paired t-test with Holm correction for multiple comparisons.

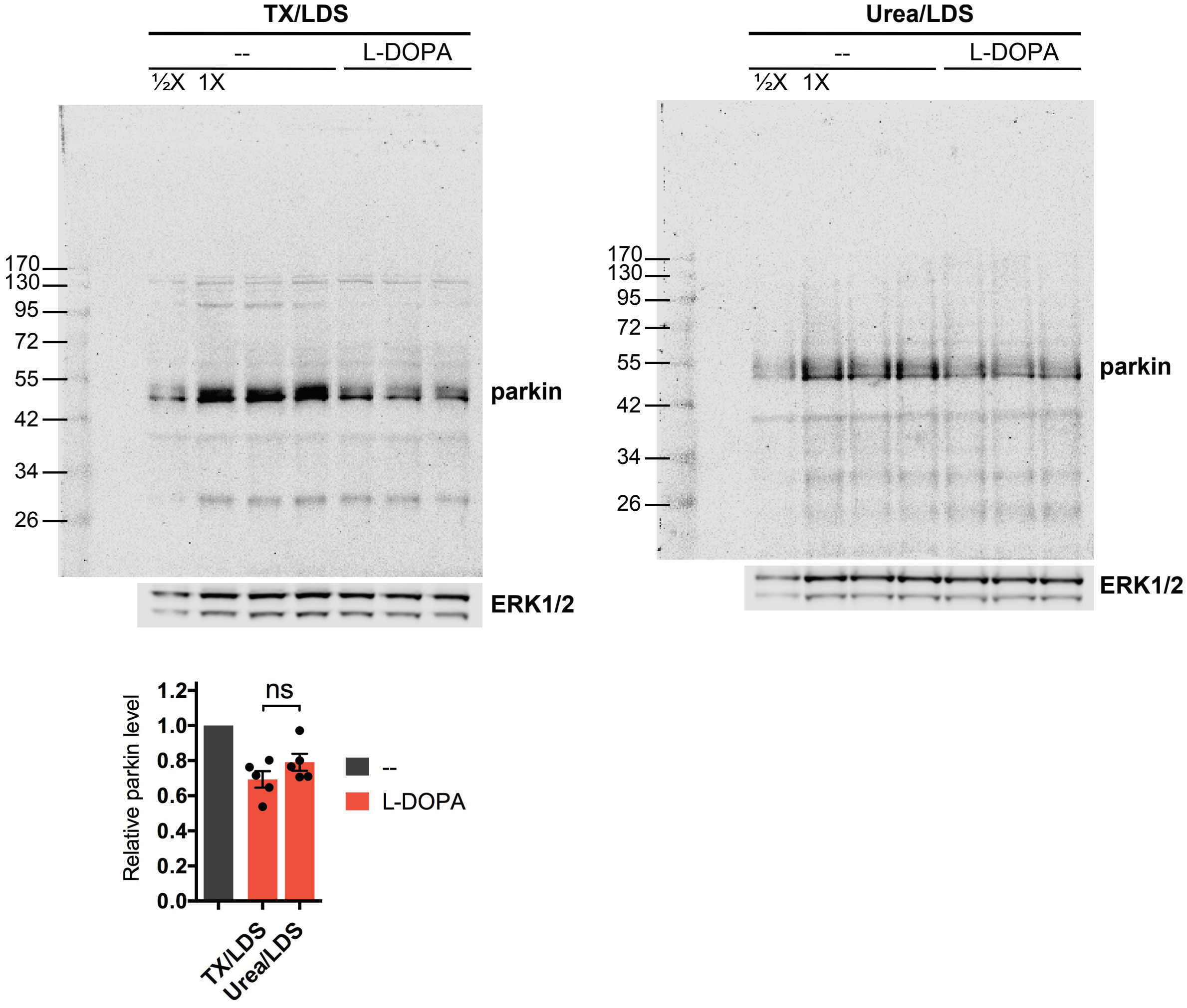

Supplementary Figure 10. L-DOPA-induced parkin loss is not attenuated by harsher cell lysis

Parkin is lost to the same extent after L-DOPA exposure whether cells are harvested in buffer containing Triton X-100 (TX) and 2% LDS (left immunoblot) or 8 M urea and 2% LDS (right immunoblot). Differentiated (N = 3) or undifferentiated (N = 2) PC12 cells were treated with 200 (N = 4) or 250 (N = 1) µM L-DOPA for 24 hours before being harvested in buffer containing Triton X-100 or 8 M urea, sonicated, supplemented with LDS and DTT, and subjected to Western immunoblotting. Parkin levels in L-DOPA-treated samples for each buffer were normalized to parkin levels in control samples in the same buffers; the gray bar in the quantification represents parkin levels in the control samples for each buffer. Error bars show SEM; statistical significance was queried by paired t-test with Holm correction for multiple comparisons.

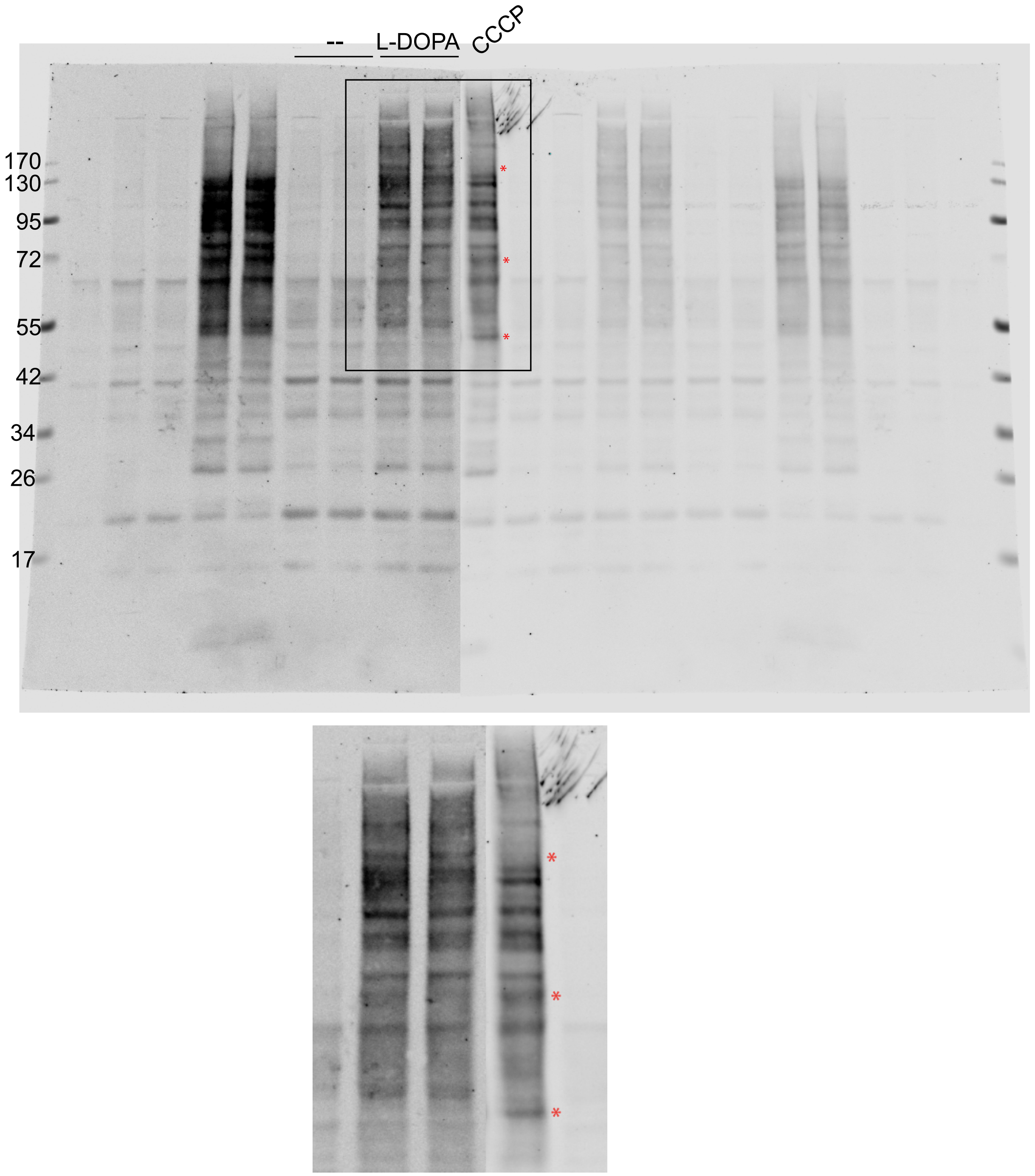

Supplementary Figure 11. L-DOPA and CCCP induce similar, but not identical patterns of phospho-poly-Ub

The Western blot in Fig. 3C was duplicated, with the exposure of one of the duplicates increased to make the L-DOPA- and CCCP-induced phospho-poly-Ub signal roughly the same intensity. The less-exposed blot was flipped horizontally, and the L-DOPA and CCCP lanes aligned for ease of comparison. Red asterisks indicate areas of difference. Area marked by rectangle is enlarged at the bottom of the image.

Supplementary Figure 12: uncropped Western immunoblots

Fig. 1a

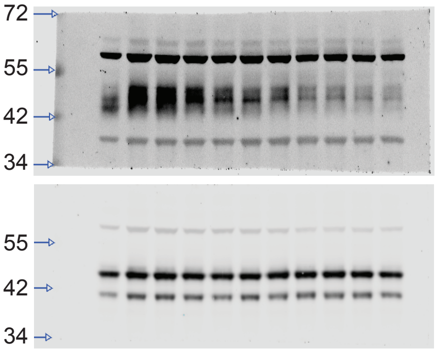

Fig. 1b

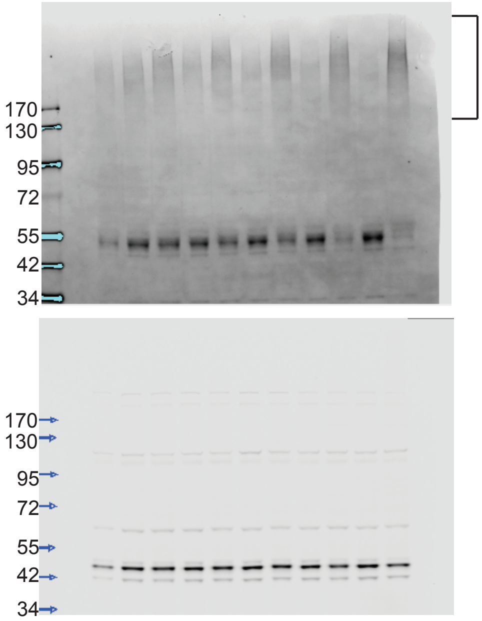

Note that these dark streaks are non-specific, appearing upon incubation of blots with secondary antibody alone in the absence of primary antibody. Additonally, parkin knockdown does not diminish their intensity.

Fig. 1c

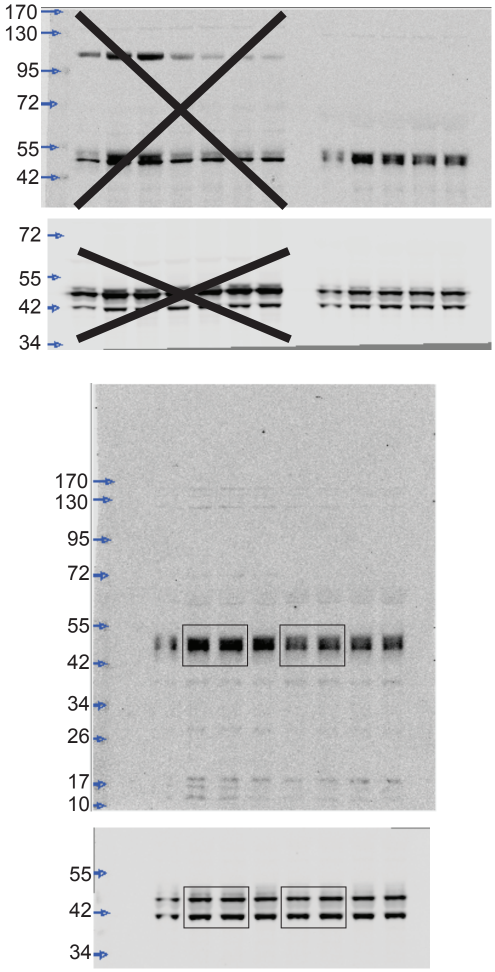

Fig. 1d

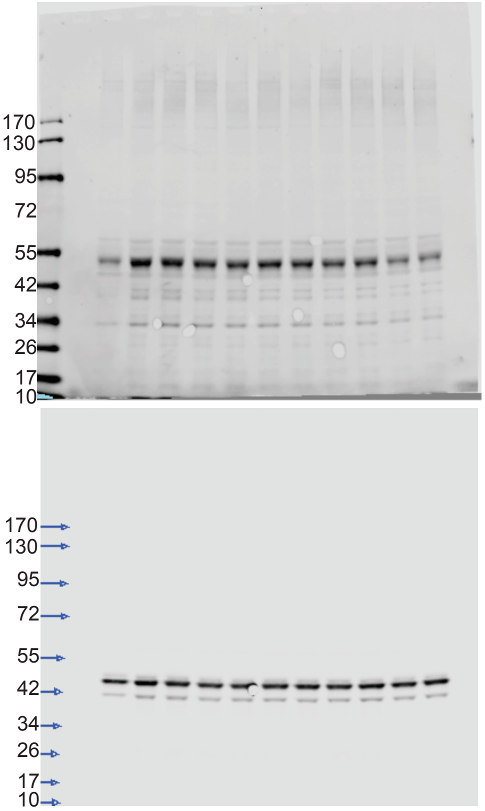

Fig. 2a

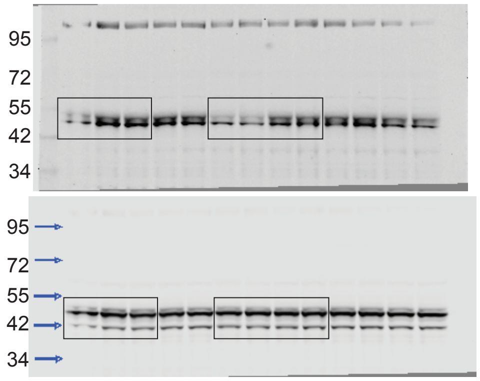

Fig. 2c

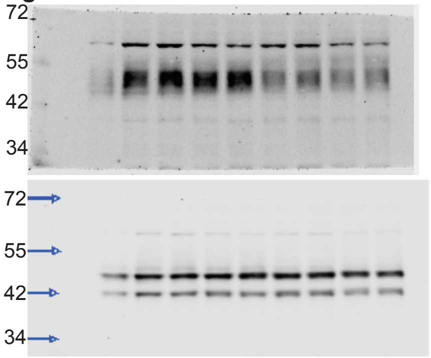

Fig. 2d

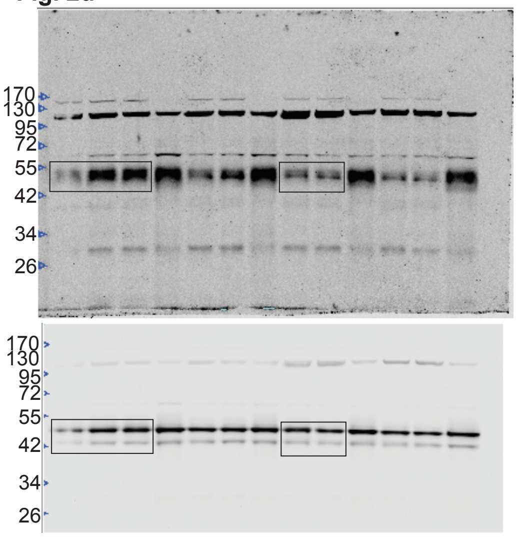

Fig. 3b

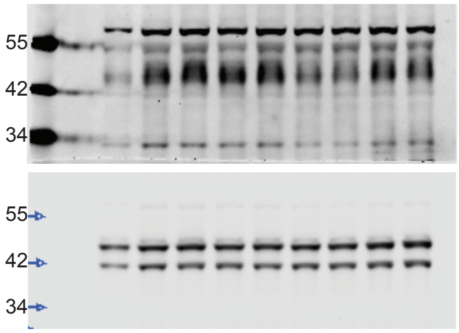

Fig. 3c

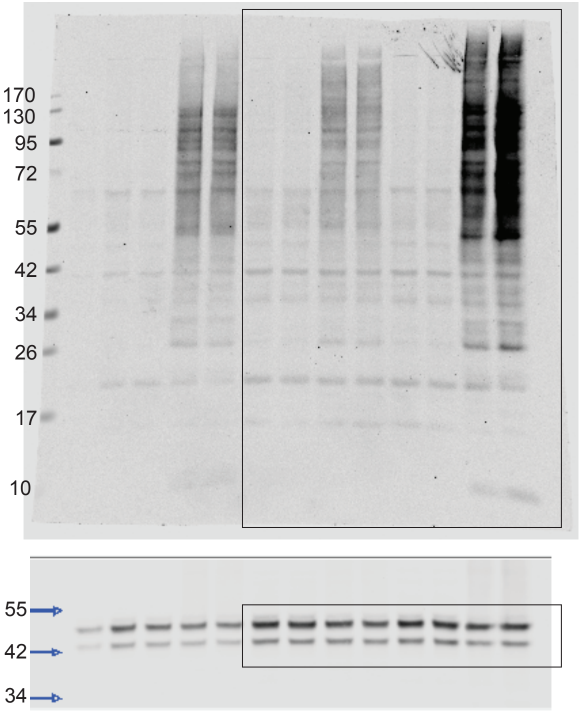

Fig. 3e

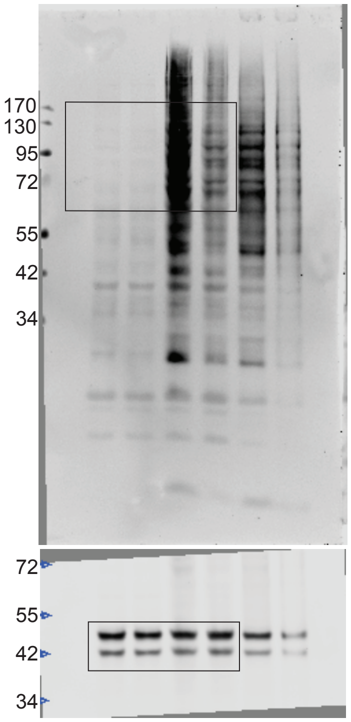

Fig. 3f

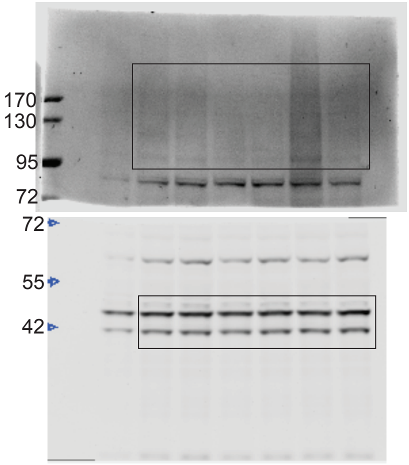

Fig. 4a

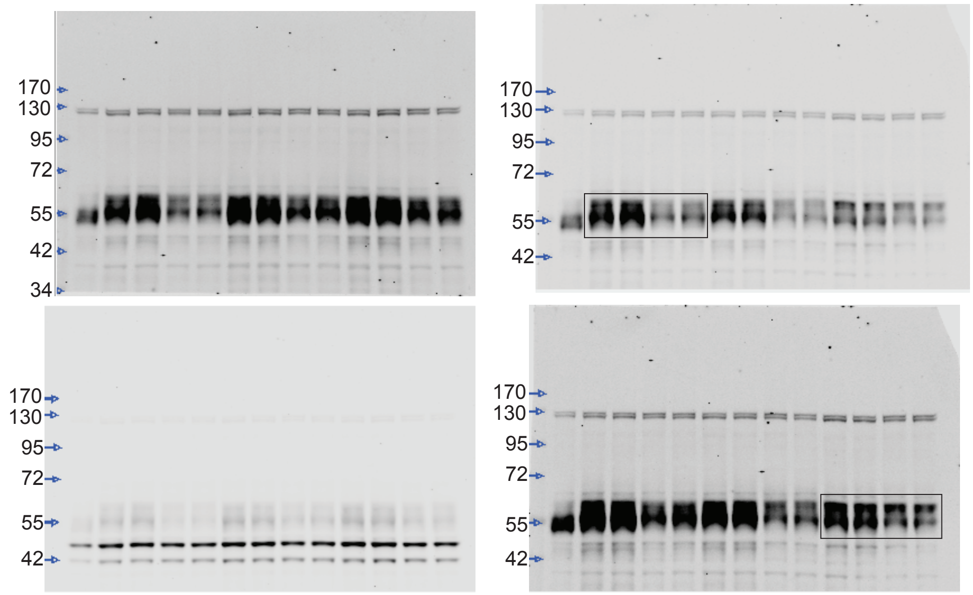

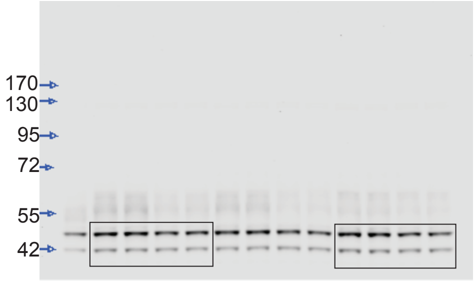

Fig. 4c

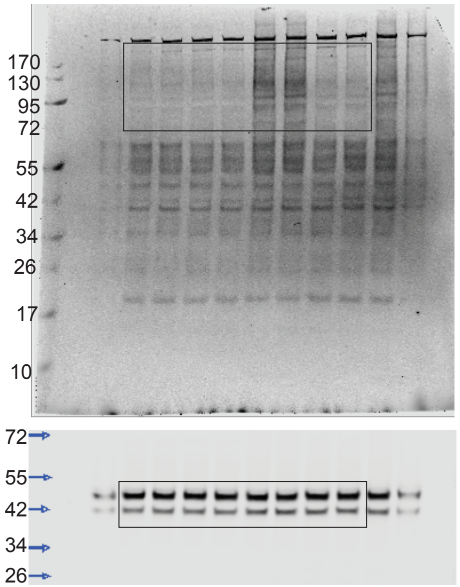

Fig. 4d

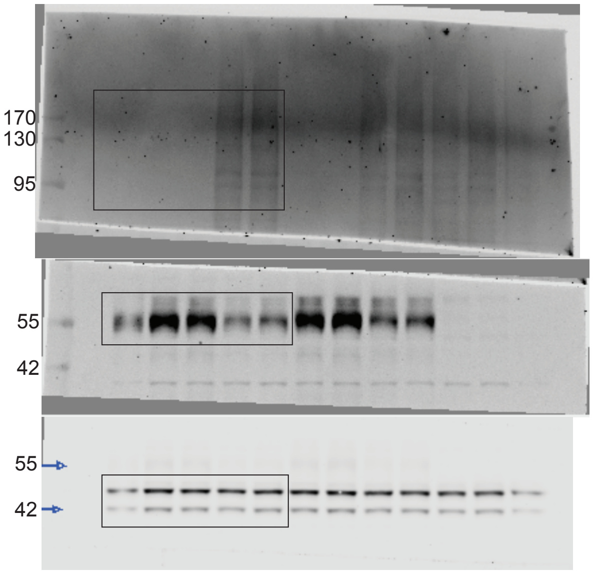

Fig. 4e

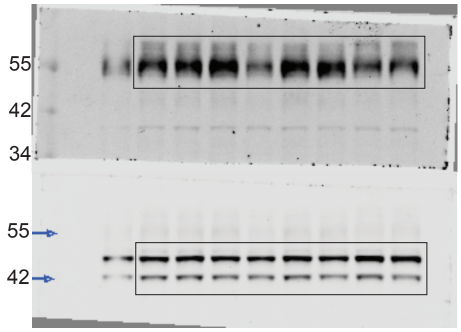

Fig. 5a

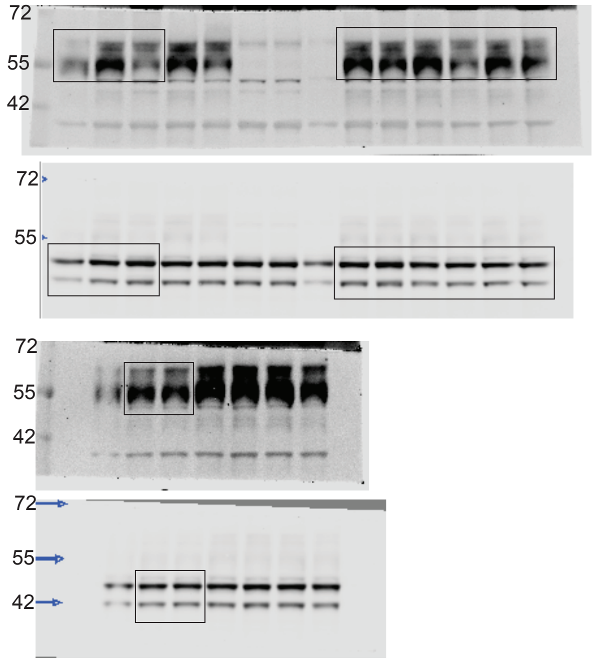

Fig. 5b

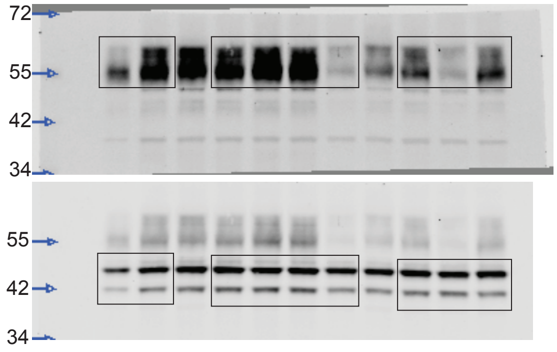

Fig. 5b, cont'd

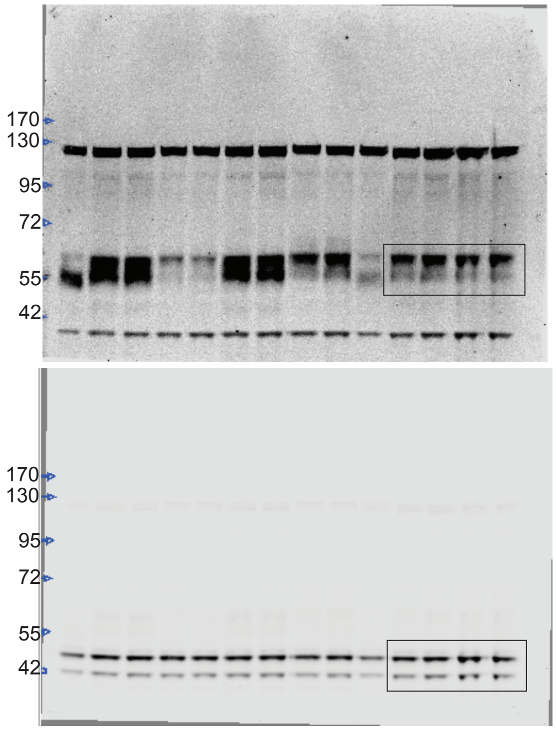

Fig. 6a

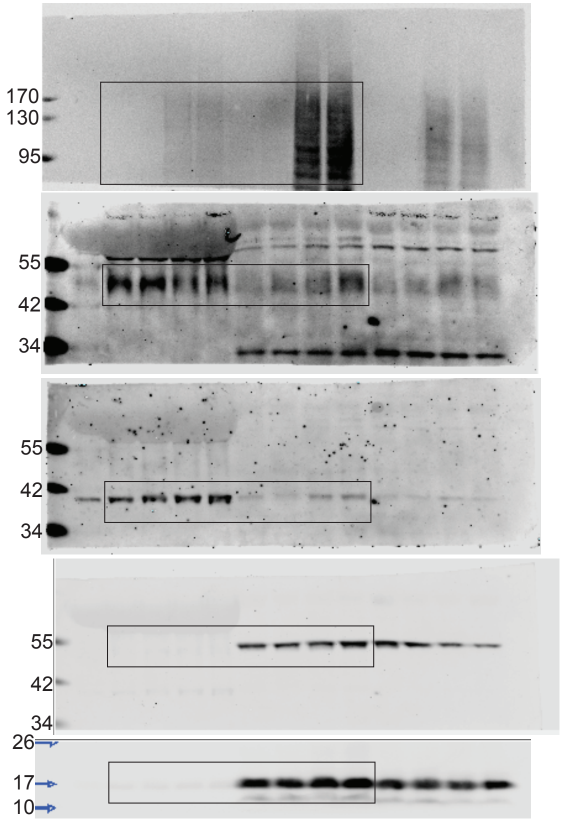

Fig. 6b

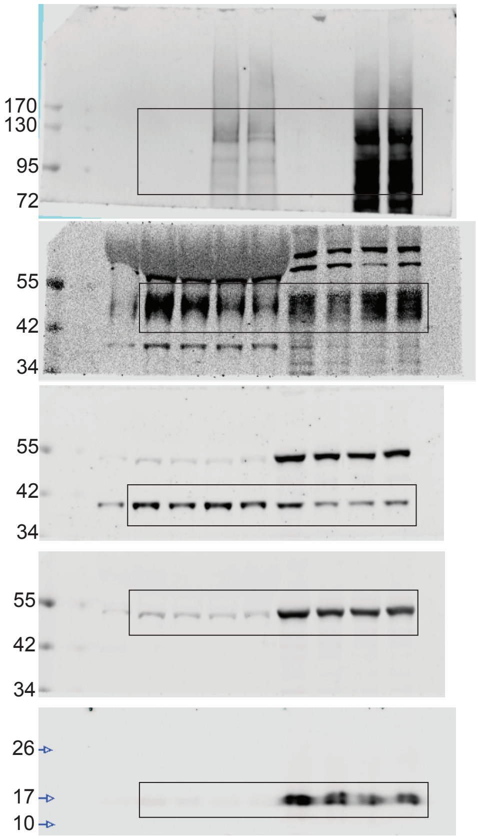

Fig. 7a

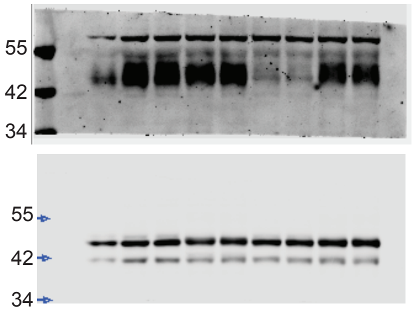

Fig. 7b

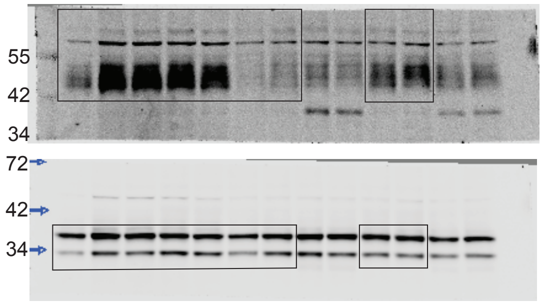

Fig. 8a

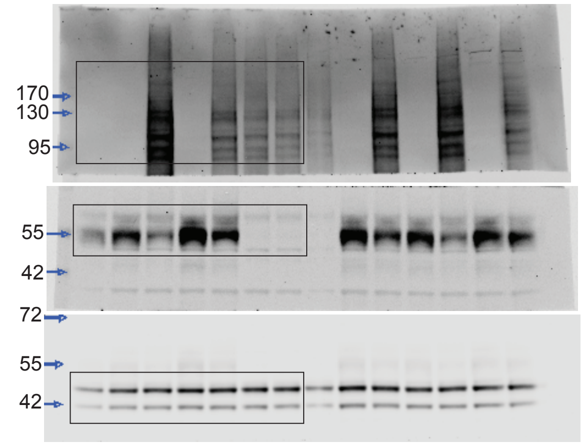

Fig. 8g

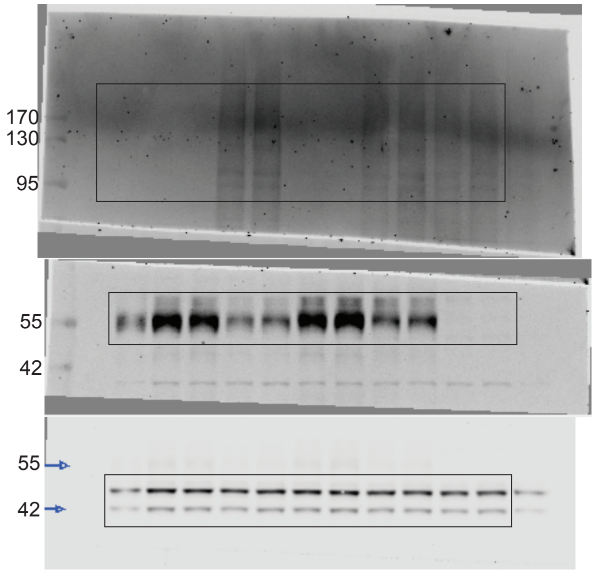

Fig. 8d

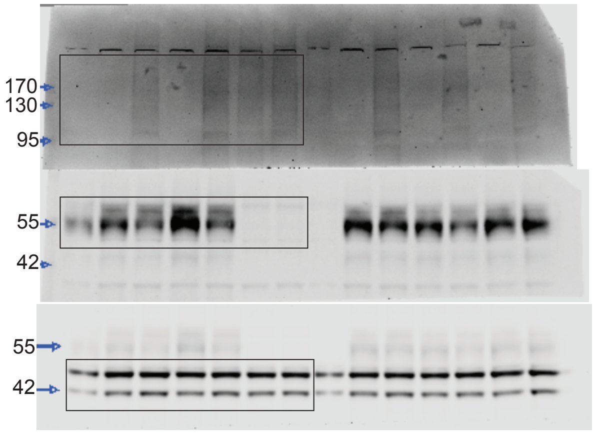

Fig. 9a

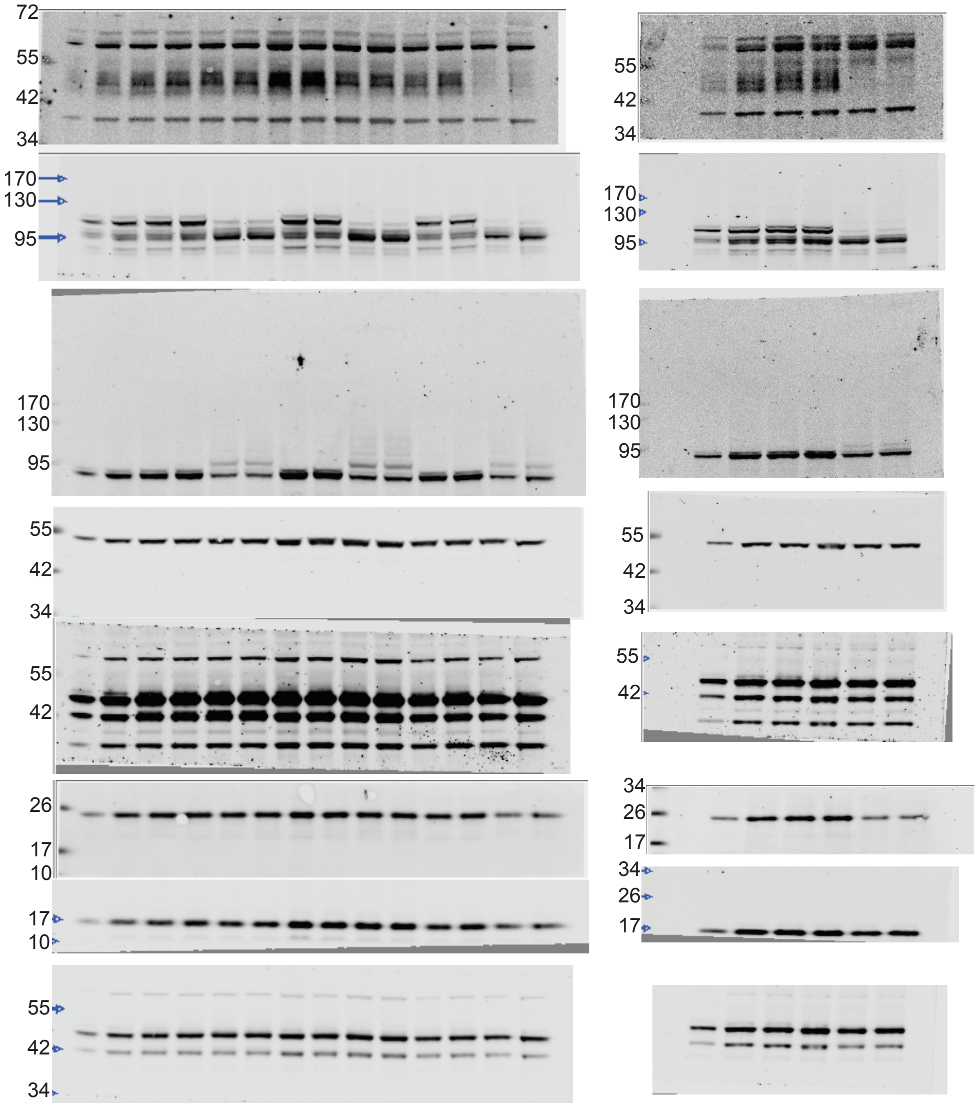
72
55
42
34
170
130
95
170
130
95
55
42
34
55
42
26
17
10
17
10
55
42
34
55
42
34
170
130
95
170
130
95
55
42
34
55
42
34
26
17
34
26
17

Fig. 9b

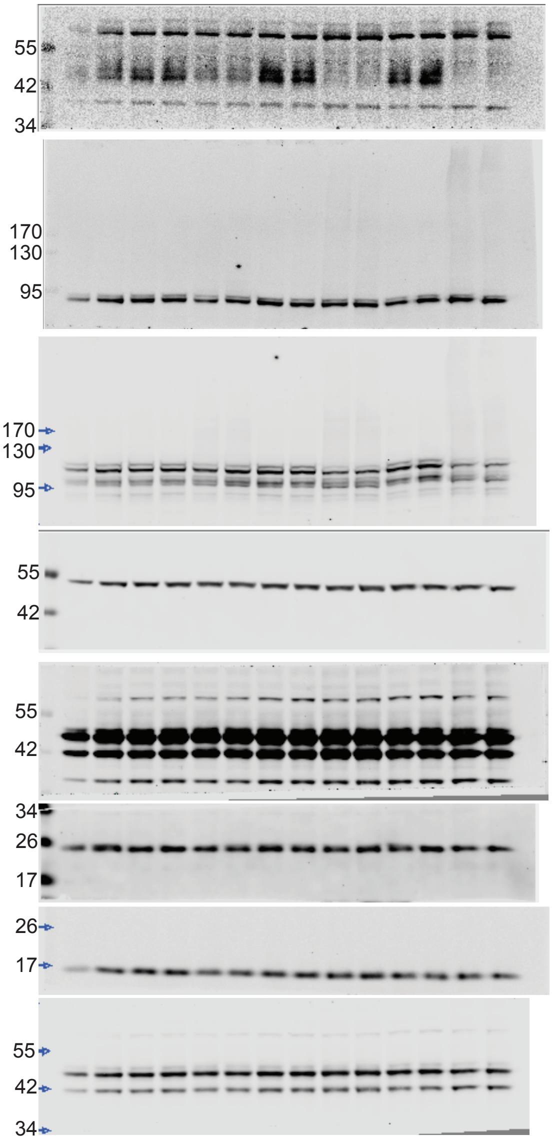

Fig. 10

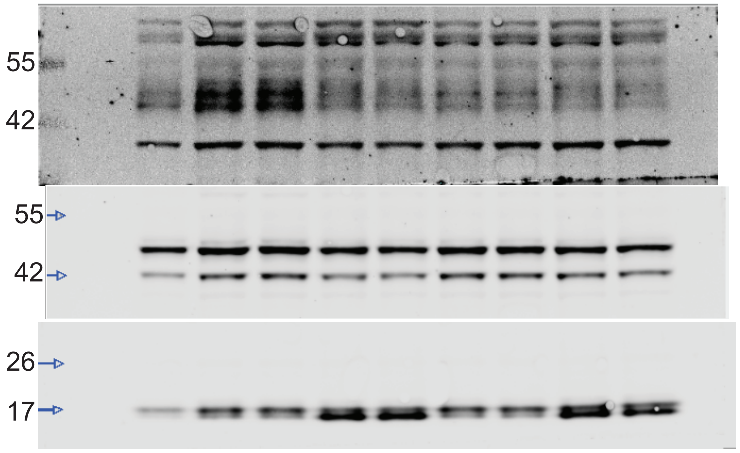

Supplement: Supplementary file 1 — Supplementary Figures [file 41598_2019_47952_MOESM1_ESM.pdf]
